# A reducing trend of fasciolosis in slaughtered animals based on abattoir data in South of Iran

**DOI:** 10.14202/vetworld.2017.418-423

**Published:** 2017-04-20

**Authors:** Manoochehr Shabani Kordshooli, Kavous Solhjoo, Belal Armand, Hamidreza Dowlatkhah, Masoud Esmi Jahromi

**Affiliations:** 1 Department of Parasitic Disease, Zoonoses Research Center, Jahrom University of Medical Sciences, Jahrom, Iran; 2 Department of Parasitology and Mycology, Jahrom University of Medical Sciences, Jahrom, Iran; 3 Department of Parasitic Disease, Research Center for Non-Communicable Diseases, Jahrom University of Medical Sciences, Jahrom, Iran; 4 Jahrom City Veterinary Offices, Jahrom, Iran

**Keywords:** fasciolosis, livestock, prevalence, south of Iran, trend

## Abstract

**Aim:**

Fascioliasis is a zoonosis infection caused by the liver trematodes (*Fasciola* spp.) which have been considered to be an important disease in livestock. After several large outbreaks, fascioliasis remains one of the serious health concerns of Iran. This study was conducted to evaluate the prevalence and possible trends of fascioliasis in slaughtered animals in South of Iran based on abattoir data during a period of 5 years.

**Materials and Methods:**

The daily records for cattle, sheep, and goats slaughtered in the abattoir were extracted from the archived documents of the recent 5 years (2011-2015) and used as the source of data. The collected data were statistically analyzed for finding any probable correlation between the various factors associated with fasciolosis.

**Results:**

Our results showed that 3.44% of all slaughtered animals during 2011-2015 were infected with *Fasciola* spp. The mean prevalence of fasciolosis for cattle, sheep, and goat was 11.15%, 5.22%, and 2.15%, respectively. In addition, the highest infection rate was in winter (4.02%), and the lowest were entered in summer (2.86%).

**Conclusion:**

Our findings showed a reducing trend during the 5 years. Improving the animal husbandry and increasing the awareness through fasciolosis may be a logical explanation for this trend. Since there have been suggested numerous factors associated with the epidemiology of fasciolosis, further studies seem essential for better clarifying the various aspects of fasciolosis in areas.

## Introduction

Fascioliasis is an infection caused by the liver fluke *Fasciola* spp. which is traditionally considered as an important disease in livestock. A wide range of mammals is known as definitive hosts for these parasites such as camel, sheep, goats, and cattle which are the most frequent animals in human environment [1,2]. These parasites cause biliary cirrhosis in livers and may cause economic losses like diminution of milk and meat production and many disorders such as diarrhea, loss of weight gain, abdominal pain, anemia, and cachexia in infected animals [3-5]. Although fascioliasis is a well-noted veterinary problem throughout the world, recent studies report it as an important public health problem as well [6-11]. Human fascioliasis is traced in 51 countries of the five continents [10,12]. In addition, many human infections may be misdiagnosed or simply not diagnosed, and hence, the number of human cases is likely greater than that is indicated by published data.

Iran is worth mentioning in this regards because of great health problems caused by fascioliasis [6,13-15]. Infection in animals and human is reported throughout the country [16-18]. As a significant example, in 90s, a large fascioliasis outbreak, including thousands of human cases, were reported in the north of Iran [19-23]. The second outbreak occurred 10 years later, and several thousand people were infected [24].

Data on the prevalence of fasciolosis and its veterinary and economic importance are scarce. In the absence of statistically and epidemiologic data, evaluating liver fluke prevalence in livestock based on abattoirs statistics might be useful. Information related to fascioliasis in different animals in middle east area were reported from some countries such as Iraq [25], Pakistan [26], Saudi Arabia [27], and Turkey [28]. Likewise, some studies published on the prevalence of fascioliasis from several regions of Iran [29-32].

Traditional and non-standard animal husbandry and the presence of large snail population are suggested to be important in the distribution of the disease in different parts of Iran. After the large outbreaks in 90s and 2000s, fascioliasis has been considered as one of the serious health concerns of the area and exerted heavy impact on the economy and social welfare of the people in Iran. Obtaining epidemiological data seem essential before managing any control programs. Information resulting from slaughterhouses records has been known as useful sources of data for the study of epidemiological aspects of certain diseases [33,34]. Therefore, this study was conducted to evaluate the prevalence and possible trends of fascioliasis in common meat production animals slaughtered in Jahrom district (south of Iran) based on abattoir data over a period of 5 years.

## Materials and Methods

### Ethical approval

The experiment on animals including all procedures of this study was approved by the local Ethical Committee in Jahrom University of Medical Sciences

### Study area

The present study is a retro-perspective study conducted at the main slaughterhouse of Jahrom region which is located in south of Iran. Geographically, Jahrom district is located between 28.19° and 29.10° latitude north and 52.45° and 54.4° longitude east. Jahrom is situated in a zone with 1050 m height from sea level, with the vast citrus gardens, where the mean monthly temperature is 21°C. However, during the warmest period (June-August) the mean average temperature goes up to 40°C, during the cooler months (December-February) the temperature drops to below 0°C. The region has a relatively poor rainfall patterns and receives around 250 mm of rainfall annually.

### Animals

The study was a retrospective abattoir survey, undertaken for 5 years from March 2011 to April 2015 which included cattle, goat, and sheep slaughtered at Jahrom main abattoir. Animals were brought there from different parts of Jahrom region. These places were in different geographical locations but more or less with the same climatic conditions, and rose with the similar animal husbandry method.

All daily records for cattle, sheep, and goats slaughtered in the main abattoir of Jahrom district were extracted from the archived documents of the recent 5 years and used as data for further evaluation. The information was collected on a monthly basis to indicate any seasonal trends.

### Statistical analysis

For clarifying the factors associated with the rate of fasciolosis, the collected data were statistically analyzed using SPSS software. The p<0.05 considered statistically significant.

## Results

In total, 190,277 animals (cattle 12,079, sheep 44,191 and goats 134,007) were slaughtered in Jahrom District during 2011 to 2015. Overall, 3.44% of all animals’ liver was condemned because of infection with *Fasciola* spp.

Among the animals, fasciolosis was detected in 11.15% of cattle, 5.22% of sheep, and 2.15% of goat. In this regard, analyzing the data related to the rate of infection between different animal showed that there was highly significant difference in *Fasciola* spp. infection within animals (p<0.001), in which infection of cattle was considerably higher than sheep and goats (Table-1).

**Table-1:** Prevalence and seasonal distribution of *Fasciola* spp. infection in different animals slaughtered in Jahrom, South of Iran, 2011-2015.

Animals	Number of examined	Number of infected (%)	95% Confidence interval	

			Lower bound	Upper bound
Sheep	44,191	2310 (5.23)	5.2103	5.2443
Goat	134,007	2887 (2.15)	2.1490	2.1597
Cattle	12,079	1347 (11.15)	11.0598	11.2433
Seasons				
Spring	44,833	1378 (3.07)	3.0442	3.1030
Summer	47,967	1371 (2.86)	2.8382	2.8866
Autumn	45,835	1719 (3.75)	3.7203	3.7761
Winter	51,642	2076 (4.02)	3.9938	4.0423
Total	190,277	6544 (3.44)	3.4258	3.4526

**Figure-1:**
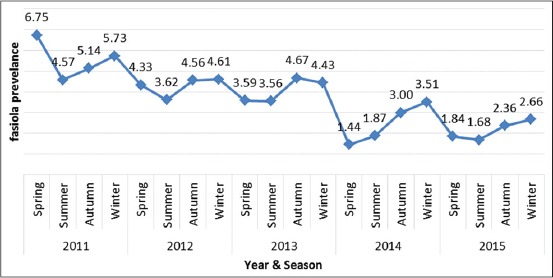
Seasonal trend of *Fasciola* spp. infection in animals slaughtered in Jahrom, South of Iran, in each years of 2011-2015.

The seasonal pattern for liver condemnation during the 5 years is illustrated in Figure-1. Briefly, the maximum and minimum rate of fasciolosis was seen in winter (4.02%) and summer (2.86%), respectively (Table-1). Comparing the result of infection rates showed that there was statistically significant difference with respect to season (p<0.005).

**Figure-2:**
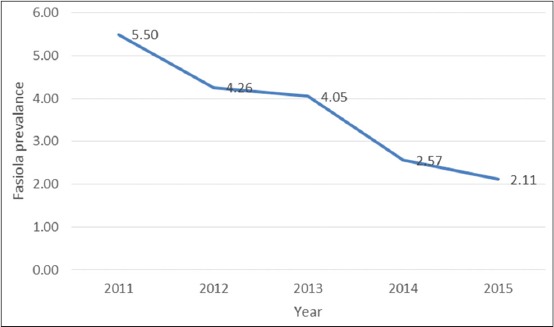
Annual trend of *Fasciola* spp. infection in animals slaughtered in Jahrom, South of Iran, 2011-2015.

As it is shown in Figure-2, annual trend of infection showed a gradual reduction from 5.5% infection rate in 2011 to 2.11% in 2015, which was statistically significant.

**Figure-3:**
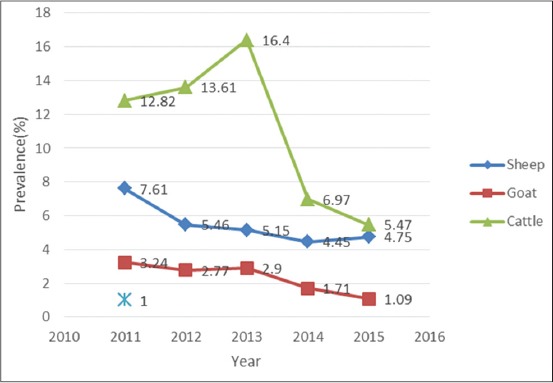
Annual trend of *Fasciola* spp. infection in different animals slaughtered in Jahrom, south of Iran, 2011-2015.

In addition, regarding the different animals, a gradual decline was seen in the infection rate of *Fasciola* spp. in goat and sheep. In cattle, the rate of infection raised at the first years, followed by a remarkable diminution in the next years (Figure-3).

## Discussion

The prevalence rate of liver trematodes in ruminants varies markedly worldwide. *Fasciola* spp. are frequently found in herbivores in different parts of Iran [13,35]. Several large epidemics of fasciolosis, including thousands of human infection, were reported from north of Iran [24,31]. In addition, another epidemic was observed in Kermanshah (west of Iran) in 2000 [36].

In this survey, 3.44% of all slaughtered animals during 2011-2015 were infected with *Fasciola* spp. In other words, the mean prevalence of *Fasciola* spp. was 11.15%, 5.22% and 2.15% for cattle, sheep, and goat, respectively.

The previous studies carried out in various regions of Iran depicted different prevalence rates of *Fasciola* spp. in livestock. A study conducted in Lorestan (southwest of Iran) indicated prevalence rate of fasciolosis among sheep and goat as 7.1% and 3.9%, respectively [37]. Similarly, Movassagh showed that 8.57% of sheep livers were polluted by *Fasciola* *hepatica* in the Northwest of Iran [38]. Daryani observed that 25.9% of cattle, 5.3% of sheep, and 4.9% of goat were infected by *Fasciola* spp.; which is compatible with our findings in Jahrom about sheep and goat, whereas the result of cattle was higher than our results [29]. Moreover, an abattoir survey in Ardabil showed that among liver flukes infections, fasciolosis was detected in 25.6% of cattle and 21.6% of sheep [39].

In the vicinity of Iran, there were reports of variable fasciolosis rate in ruminants. In Pakistan (east of Iran), a considerably high prevalence rate of *F. hepatica* in cattle (85.1%), sheep (51.3%) and goats (14.8%) was reported [40]. In contrast, a very low prevalence was observed in Iraq, where an abattoir data indicated that fasciolosis rate in cattle, sheep and goat was 0.13%, 0.72%, and 3.30%, respectively [41]. Furthermore, fasciolosis has been reported in 3.99% of sheep and 0.48% of cattle in Turkey [28].

The differences observed could be explained through the existence of various factors such as husbandry practices and climatic variation. Rainfall and temperature were among those climate items considered as effective factors in the distribution of fasciolosis among herbivores [42]. A probable reason could be the fact that rainfall and temperature had a significant impact on the survival of both intermediate host and the larval stages (miracidium and cercariae) of the parasite [43].

In this regard analyzing, the data related to the prevalence of fasciolosis in different season indicated that there was a distinct correlation between season and fasciolosis among animals. As indicated in Table-1, liver condemnations due to fasciolosis were more prevalent in animals slaughtered during winter, whereas the lowest rate of infection was seen in summer. The higher rate of fasciolosis during winter might be correlated with the above mentioned meteorological factors, in which higher rainfall in winter probably caused the higher population of snails and consequently increasing the infection in animals. Moreover, it could be hypothesized that the reduction of infection in animals accompanied by very hot and dry environmental condition at summer [30]. A distinct seasonal pattern for fasciolosis among herbivores was reported in most of epidemiological surveys [37,44-48]. All studies had a common point that, the rainier the season, the more fasciolosis infection indicated.

These are other factors contributing to the epidemiological aspects of fasciolosis. For example, certain kinds of soil affect the distribution of intermediate host in environment [49,50]. Regardingly, all criteria related to soil, including minerals, porosity, pH and other physical and chemical characteristics of the soil are deemed to be important in this subject [51,52]. This indicates the complexity of the various aspects of fasciolosis cycle, which suggests for further work to properly determine all the possible factors associated with the fasciolosis prevalence.

Our findings were also in agreement with that of those who find a higher rate of infection in cattle compared to sheep and goat [3,29]. Similar results were also reported in Kenya where a considerable rate of fasciolosis was seen in cattle (52.6%) compared to sheep (18.3%) and goat (16.9%) [53]. In a 10 years abattoir investigation in Khuzestan province, higher fasciolosis prevalence was observed among cattle (5.54%), more than sheep (0.93%), and goat (2.79%) [30].

On the other hands, Talari, in a 3-year abattoir investigation, reported a lower prevalence of fasciolosis in cattle (2.4%) than sheep (6.9%) and goat (4.1%) which was clearly contrary to our results [44].

Moreover, the previous studies indicated that fasciolosis rate in sheep was significantly higher than goat [3], which was compatible with the results of our study.

An abattoir data survey of slaughtered animals of Mazandaran (north of Iran) revealed that 5.7% of sheep and 1.6% of goats were infected with *Fasciola* spp. [31].

The epidemiologic notion of this finding might be attributed to the grazing style of animals on grassland. Cattle usually tend to pasture near the springs and streams, where the snails are more frequently present. In contrast, goats usually have the lowest rate of infection compared to sheep and cattle. The grazing style of goat is different from that of sheep, in a way that they naturally tend to eat leaves and heaths in elevated areas, contrary to sheep that often graze on the land; this way of pasturing may reduce the contact with infective metacercariae and more probably reduces the risk of infection in goat.

It is worth to mention that our findings showed a gradual reduction of fasciolosis from the beginning of 2011 to the end of 2015. In cattle, the story was a bit different: The graph showed a raising trend at the first years, following by a steep slope of diminution at the end of the period. The animal husbandry method could explain the variation observed in the distribution of fasciolosis in different regions. After several epidemics in Iran, the awareness among farmers to standardize the method of husbandry was remarkable. Moreover, effective using of available treatments (triclabendazole or albendazole) has been widely developed in recent years that brought about reduction in all types of veterinary parasites including fasciolosis [30]. Cattle husbandry has dramatically changed during the years. Today, cattle usually are kept in modern farms with less contact with environment, and this has led lower infection in animals compared to the past.

## Conclusion

Regarding the foregone discussion, we observed a relatively low prevalence and also a significant reduction of fasciolosis among sheep, cattle, and goats during the recent 5 years. A wide spectrum of factors has been suggested that affects the distribution of fasciolosis in animals and human; thus, further studies should be done to clarify the whole aspects of fasciolosis. Abattoir surveys could be helpful as it provided useful preliminary information for further monitoring. Logically, although the slaughterhouse data might not be equal to the real population of animals, it could be considered as a marker of real infection in population. In addition, it seems useful for managing and evaluating the control and prevention programs.

## Authors’ Contributions

BA and MSK have designed the concept and supervised the plan of work and also have prepared the manuscript. HD, MEJ, and MSK have contributed in data collection, administrative, technical, and material support. KS and BA have analyzed and interpreted the data.
